# Supplementary figures and images for: Chromosome-Level Genome Assemblies Expand Capabilities of Genomics for Conservation Biology
Source: Genes (Basel). 2021 Aug 28;12(9):1336. doi: 10.3390/genes12091336 (PMC8466942; doi:10.3390/genes12091336)

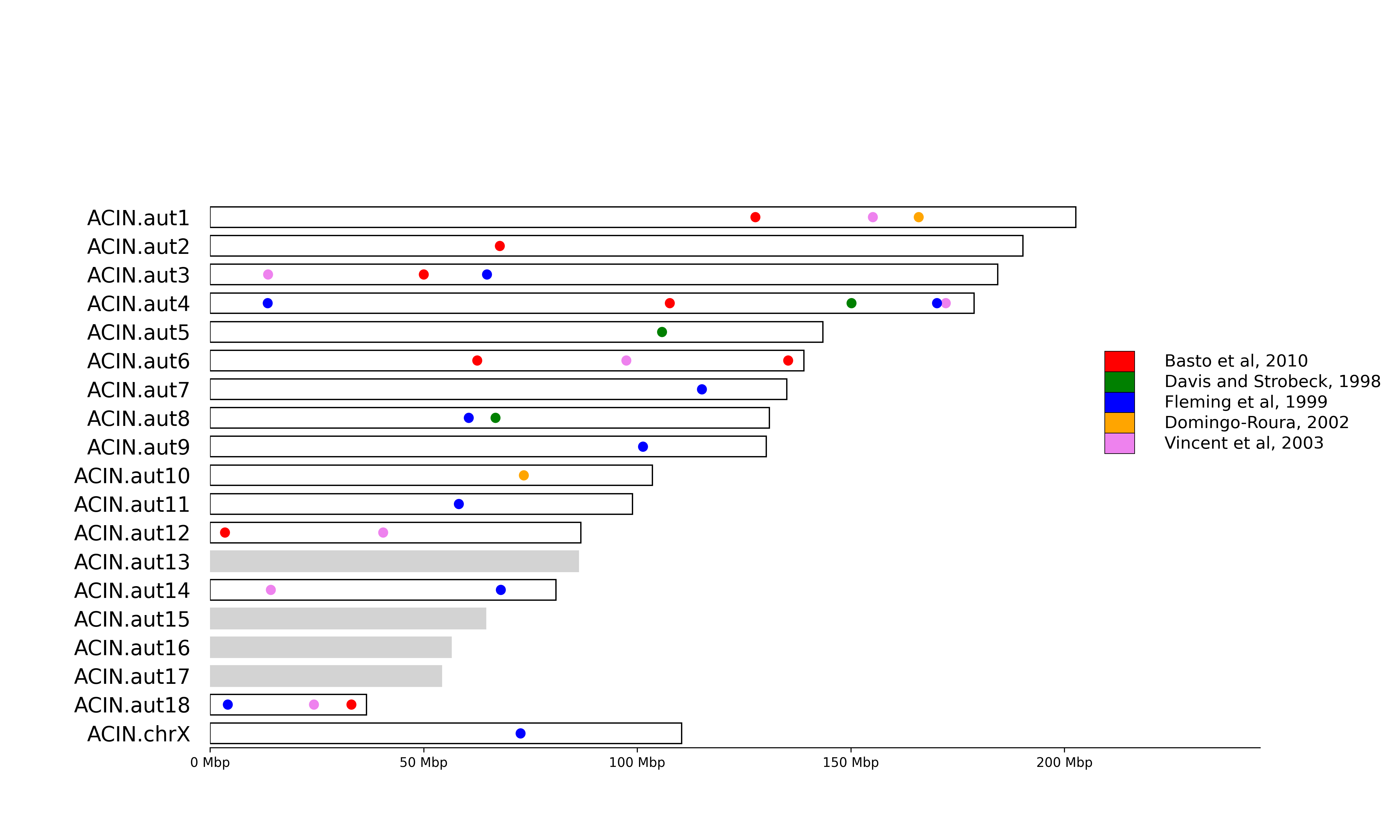

Supplement: Supplementary file 1 [file genes-12-01336-s001.zip › figures/Figure_S1.aonyx_cinereus.STR.png]

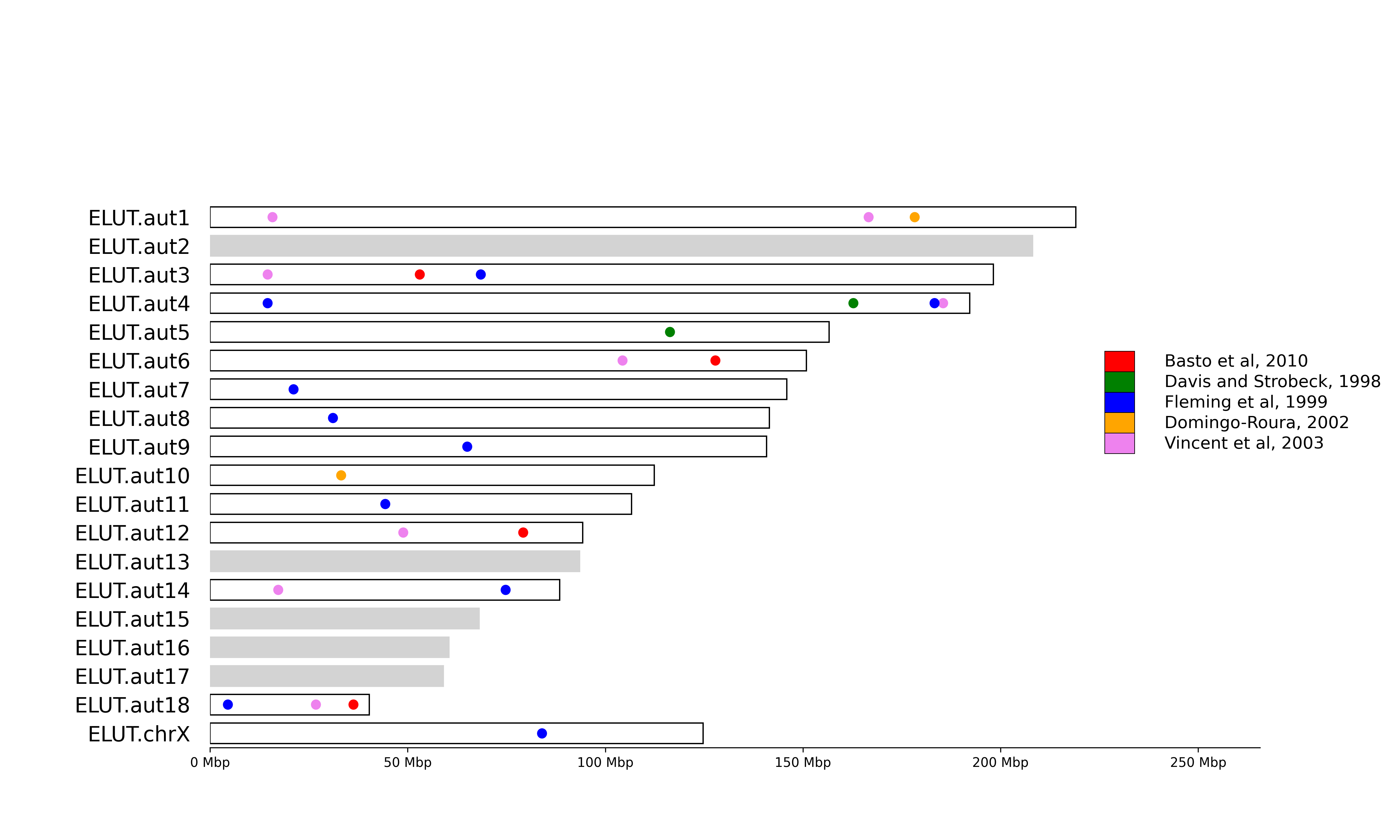

Supplement: Supplementary file 1 [file genes-12-01336-s001.zip › figures/Figure_S2.enhydra_lutris.STR.png]

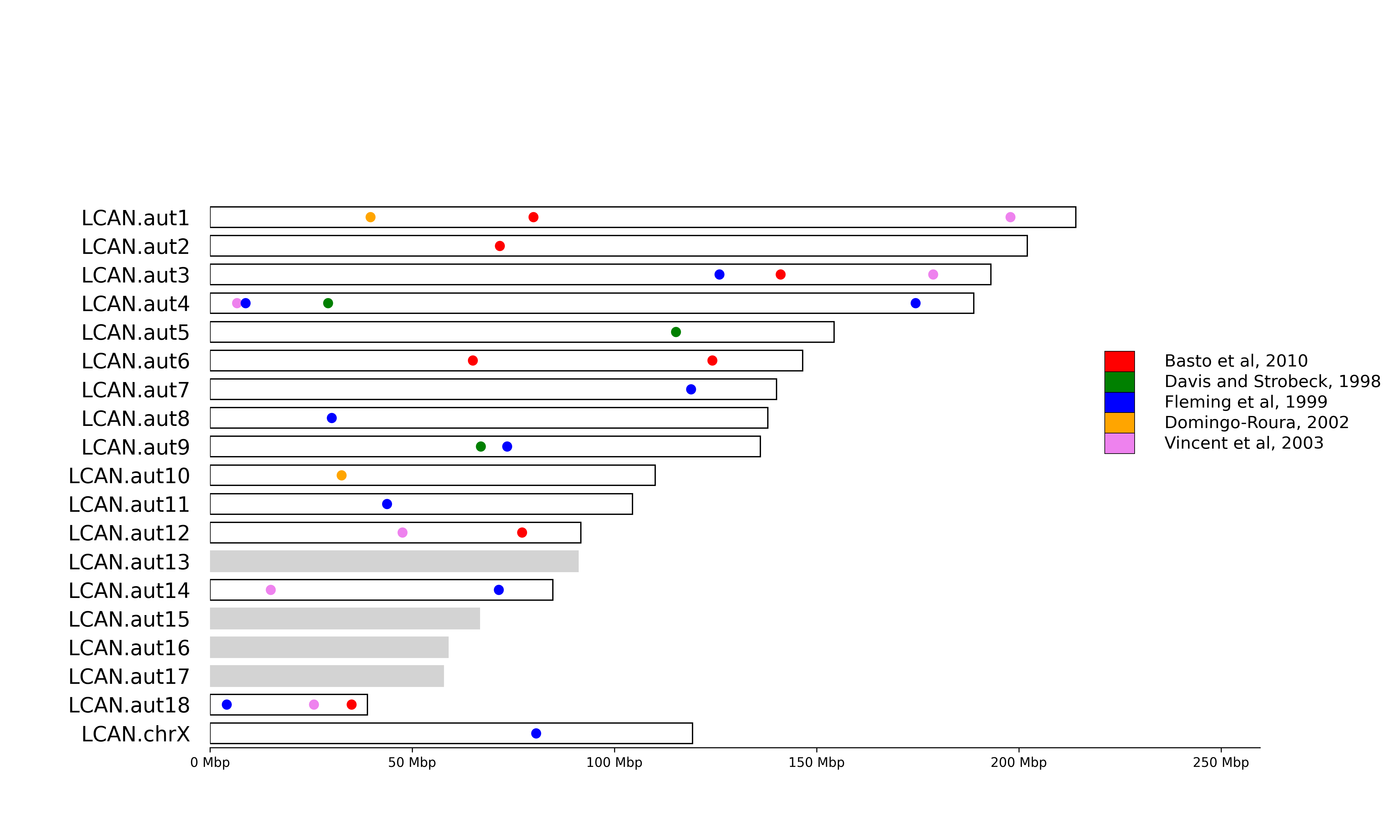

Supplement: Supplementary file 1 [file genes-12-01336-s001.zip › figures/Figure_S3.lontra_canadensis.STR.png]

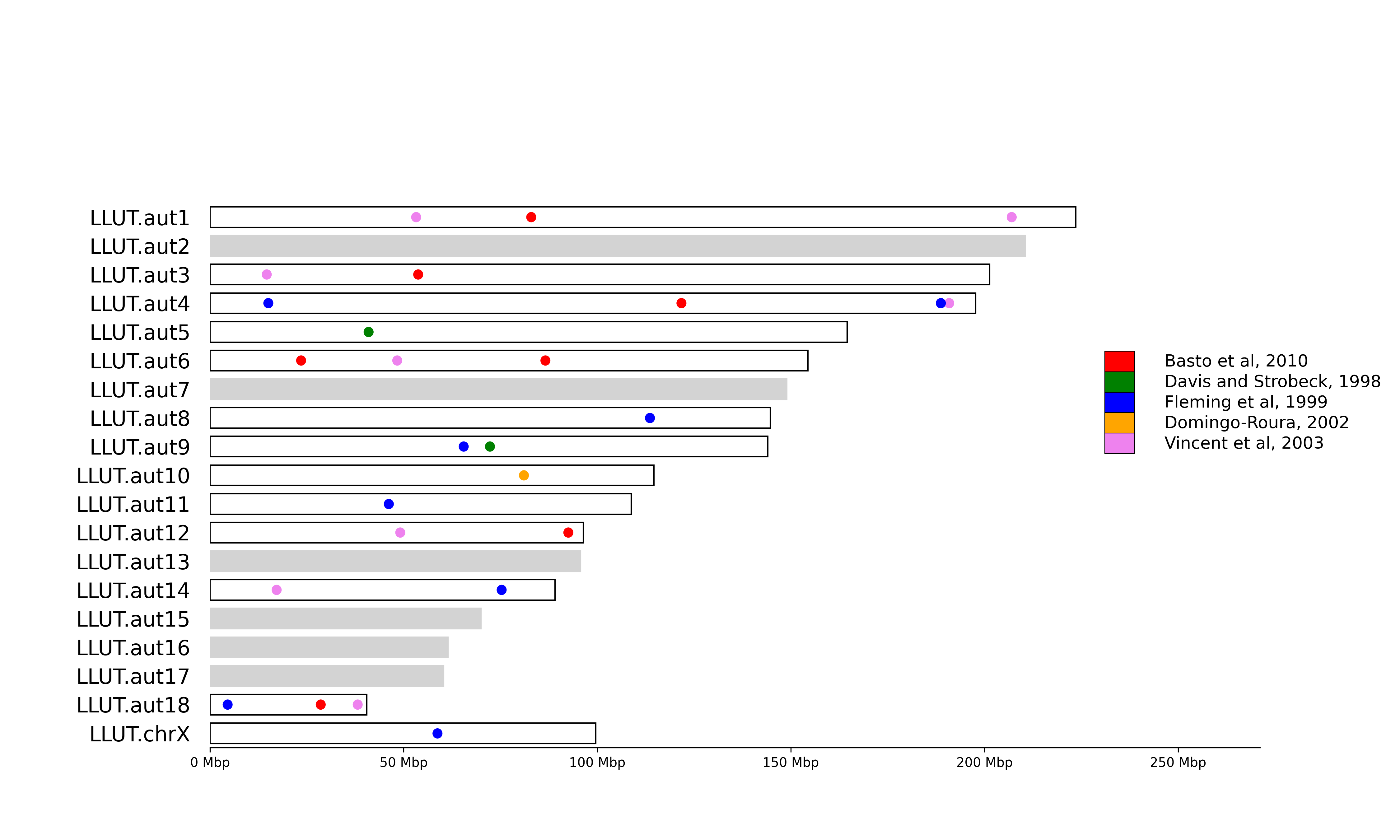

Supplement: Supplementary file 1 [file genes-12-01336-s001.zip › figures/Figure_S4.lutra_lutra.STR.png]

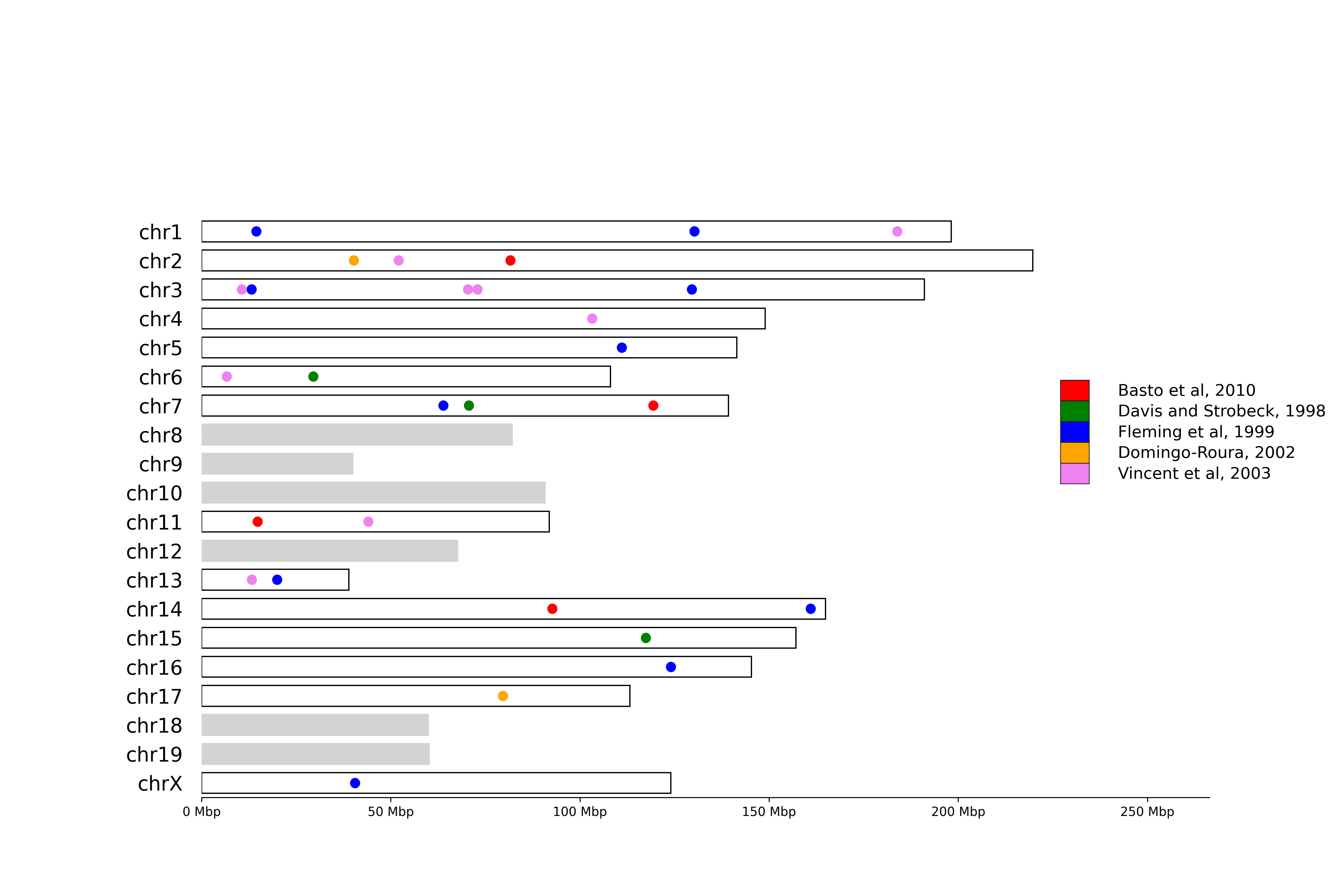

Supplement: Supplementary file 1 [file genes-12-01336-s001.zip › figures/Figure_S5.mustela_putorius_furo.STR.png]
